# Clinical Utility of Intra-Operative 6% Hydroxyethyl Starch (130 / 0.4) Supplementation in Hypoxemic Femur Injury Patients: A Preliminary Report of Twenty Cases

**DOI:** 10.5812/atr.6847

**Published:** 2012-10-14

**Authors:** Indu Sen, Vinod Kumar, Govedhan Das Puri, Ramesh K Sen

**Affiliations:** 1 Anaesthesia and Intensive Care Department, Post Graduate Institute of Medical Education and Research, Chandigarh, India; 2 Orthopedic Surgery Department, Post-Graduate Institute of Medical Education and Research, Chandigarh, India

**Keywords:** Intravenous Fluids, Trauma Surgery, Hydroxyethyl Starch

## Abstract

**Abstract:**

Posttraumatic intravasation of fat and debris can lead to a cascade of events. Hydroxyethyl starches (HES) markedly suppress neutrophil influx by decreasing pulmonary capillary permeability and facilitating tissue oxygenation by improving microcirculation. It was hypothesized that in hypoxemic femur injury patients undergoing operative stabilization, HES administration will prevent the deterioration of respiratory variables and facilitates recovery. This prospective, double-blind, randomized preliminary study, enrolled twenty posttraumatic hypoxemic patients (room air PaO2 < 70 mmHg, Schonfeld fat embolism index score (SS) > 5) scheduled for femur fracture stabilization under general anesthesia. Patients were allocated to receive either; 6% HES 130/0.42, 15 mL/kg or 0.9% normal saline (NS) to maintain their central venous pressure (CVP) 12 + 2 mm Hg. Blood was transfused according to the maximum allowable blood loss and by serial hematocrit estimations. Perioperative Glasgow Coma Scale (GCS), physiological variables, arterial oxygen saturation (SpO2), arterial blood gas (ABG), SS and P/F ratios were recorded until recovery. The partial pressure of oxygen in arterial blood / fraction of inspired oxygen ratio (PaO2/FiO2) improved from a preoperative value of 273.33 ± 13.05 to 435.70 in the 6% Hydroxyethyl starch group (HES) and from 275.24 ± 15.34 to 302.25 ± 70.35 in the NS group over a period of six days (P values =0.970, 0.791, 0.345, 0.226, 0.855, 0.083, 0.221). Time taken to achieve a P/F ratio > 300 and for persistent reduction of Murray’s lung injury score (LIS) were comparable (P = 0.755 and 0.348, respectively). The number of ventilator, intensive care unit (ICU) and hospital stay days, did not differ (P value = 0.234, 1.00, 0. 301, respectively). There were no adverse sequelae or mortalities. A trend showing relatively fast improvement in the P/F ratio and an early reduction in LIS values was observed in hypoxemic, femur injury patients receiving intraoperative colloid supplementation.

## 1. Introduction

Posttraumatic intravasation of fat and debris can lead to a cascade of events including neutrophil activation which results in acute lung injury. Various surgical and pharmacological techniques have been developed in an attempt to reduce the intravasation of fat, or to prevent alveolar damage during trauma surgery (1). Colloids with a volume effect of approximately 100% remain in circulation for four to six hours, these markedly suppress neutrophil influx by decreasing pulmonary capillary permeability and this facilitates tissue oxygenation by improving microcirculation (2-5). On the other hand, crystalloids can adversely affect pulmonary functioning by increasing closing volume, reducing static/dynamic compliance and by causing pulmonary edema (6, 7). It was hypothesized that in hypoxemic, femur injury patients undergoing operative stabilization, intraoperative hydroxyethyl starch (HES) administration would prevent the deterioration of respiratory parameters and facilitates recovery.

## 2. Main Text

**Figure 1.**
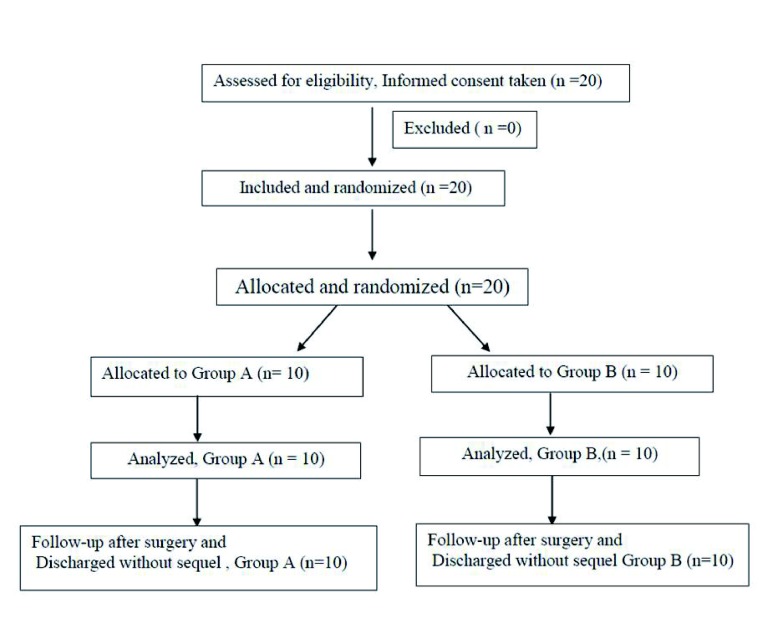
The Flow Chart of the Study Groups

After approval by the ethics committee of Post Graduate Institute of Medical Education and Research (PGIMER), Chandigarh and written informed consent, this prospective, randomized-trial enrolled twenty consecutive ASA I or II adults of either gender, aged 18 - 45 years, with acute femur injury and post traumatic hypoxemia (partial pressure of oxygen in arterial blood (PaO_2_) < 70 mm Hg, on room air and Schonfeld fat embolism index (SS) score > 5) (8), were admitted to the trauma ward of a tertiary care postgraduate research institute, Chandigarh (Figure 1).

Chest roentgenogram, 12 - lead electrocardiography, arterial blood gas analysis and Murray’s lung injury score (LIS) calculations (9) were recorded 12 hourly for all patients. Prior to surgery, serum hemoglobin > 10 gm/dL, normal serum electrolytes, coagulation profiles and hepatorenal parameters were ensured. All patients had stable hemodynamics, without ionotropic support, and LIS values between 0.5 - 2.5. Patients with associated cerebral, thoracoabdominal trauma, myocardial insufficiency (myocardial infarction (MI) or congestive cardiac failure (CCF)), diabetes mellitus, and allergy to colloids, hepatorenal disorders, pathological fractures or pregnancy were not enrolled. A standardized general anesthetic technique was used. Monitoring consisted of electrocardiography (ECG), arterial oxygen saturation (SpO_2_), invasive blood pressure (IBP), fraction of inspired oxygen (FiO_2_), airway pressures (Paw), end tidal carbon dioxide (EtCO_2_), central venous pressure (CVP) and urine output (UO). All patients were allocated to receive one of the two intravenous fluid regimens intra operatively using a computer generated permuted block randomization method (randomized blocks of four patients in a 1:1 ratio using sealed envelopes), while CVP was maintained, 10 - 14 mmHg. The hydroxyethyl starch group (n = 10) patients were administered 6% HES 130/0.4 (2-4 mL/kg/h). If CVP was < 10 mmHg, 6% HES (130/0.4) was infused at the rate of 8 - 10mL/kg, but the maximum amount of colloid administered during surgery was limited to 15 mL/kg. Patients in the 0.9% normal saline (NS) group (n = 10) were infused crystalloids intraoperatively. In all patients, whole blood was transfused when blood loss exceeded the calculated maximum allowable blood loss (MABL), or intraoperative hemoglobin estimations revealed values below 8 mg/dL. Perioperative Glasgow Coma Scale (GCS), heart rate, blood pressure, SpO_2_, arterial blood gases, SS, P/F ratio and LIS were continually recorded until the patient was taken off oxygen therapy. Lung injury scores indicate the severity of lung injury (9). It has four components: hypoxemia score, chest X-ray score, compliance score and positive end expiratory pressure (PEEP) score. The P/F ratio or oxygenation ratio quantifies the extent of lung injury. Values less than 300 indicate acute lung injury, and values less than 200 with bilateral chest infiltrates and noncardiogenic pulmonary edema, suggest acute respiratory distress syndrome. In the present study, primary outcome measures were; time to achieve PaO_2_/FiO_2_ ratio more than 300, time to achieve persistent reduction in the LIS by 0.5. Secondary outcomes were; number of patients requiring mechanical ventilation, duration of ventilatory support, length of ICU stay, hospitalization period and morbidity/mortality during hospital stay. Statistical analysis was performed. Data was explored for outliers, typing errors and missing values. To determine if there was any statistical association between the various categorical variables, a comparison of the two groups was carried out using a Chi-square test. The demographic data, duration of injury and other quantitative variables were compared using an independent t-test or Mann-Whitney U-test depending upon the distribution (symmetrical or asymmetrical). Discrete variables such as gender were analyzed by a Chi-square test. Continuous data such as heart rate, blood pressure, and CVP, were analyzed by repeated measure ANOVA, followed by one way ANOVA to assess the trend of change in the serial values and to find out the interaction of trends in the two groups. For time to event data, survival analysis using Kaplan Meier and log rank tests were carried out. Quantitative data has been reported as Mean ± SD with graphical presentation as 95% confidence interval error bars. Categorical data is shown as frequencies. The frequency distribution of the study characteristics is shown separately for HES and NS group patients. Statistical significance was defined as P value (two tailed) ≤ 0.05. 

## 3. Results

**Figure 2.**
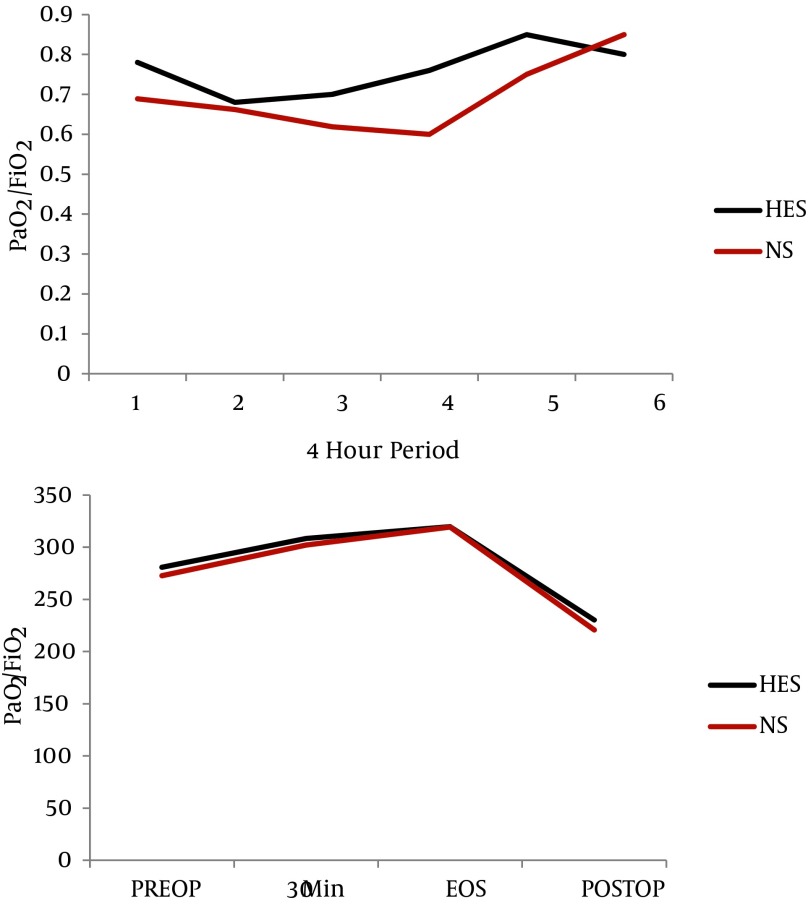
Comparison of PaO2/FiO2 Ratio at Different Time Intervals in HES and NS Group

**Figure 3.**
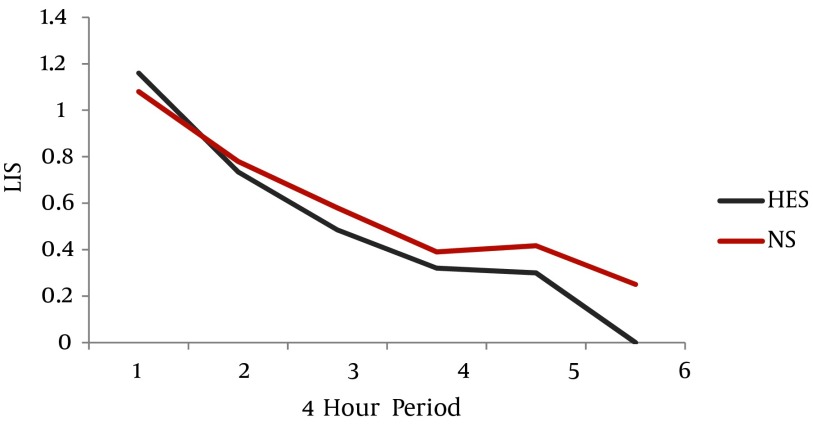
The Trend of Reduction in Lung Injury Scores (LIS) in HES and NS Group

**Figure 4.**
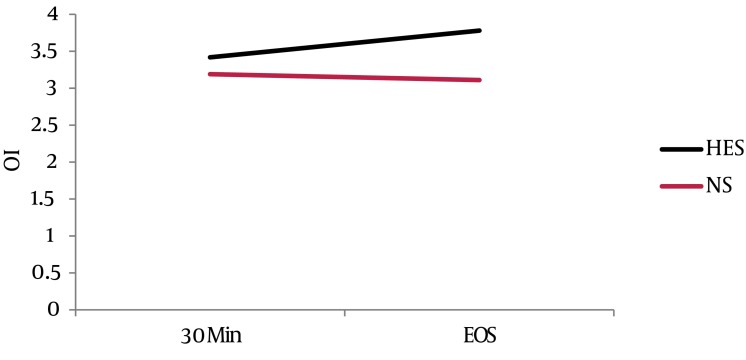
Oxygen Index (OI) at 30 Minutes of Induction and End of Surgery in HES and NS

Demographic characteristics, new injury severity score, time lapse from injury to admission and injury to surgery, duration of surgery, time of reaming and anesthesia time were similar in both groups (Table 1). Serial hematological, biochemical and arterial blood gas analysis values were statistically comparable between the two groups (*P* > 0.05). The two groups effectively maintained intraoperative physiological variables (Table 1). The mean volume of hydroxyethyl starch 130 / 0.4 infused was 1015 ± 52.96 mL in the HES group. Eight patients in the HES group required saline solution for maintenance, after the maximum limit of 15 mL/kg HES was administered intraoperatively. The mean volumes of normal saline infused in groups HES and NS were 695 ± 491.28 mL and 3600 ± 614.63 mL, respectively (*P* = 0.00). Estimated blood loss was 820 ± 154.91 mL in the HES group and 950 ± 158.11 mL in the NS group (*P* = 0.094). There were no relevant differences in the amount of whole blood transfused amongst the groups (*P* = 0.789). None of the patients required any other blood products. Urine output was adequate in both the groups and diuretics were not required. The P/F ratio increased from 280.84 ± 13.05 to 435.70 in the HES group and from 272.69 ± 15.34 to 302.25 ± 70.35 in the Normal Saline group over a period of six days (*P* = 0.970, 0.791, 0.345, 0.226, 0.855, 0.083, 0.221). The trend of improvement in the P/F ratio was faster in the HES group compared to the NS group. Time taken to achieve P/F ratio > 300, was 1.60 ± 0.31 days in the HES group and 1.80 ± 0.44 days in the NS group (*P* = 0.75) (Figure 2). LIS values were reduced from 1.16 ± 0.43 to zero, in four days and from 1.08 ± 0.51 to zero, in five days in the HES and NS group, respectively. Time taken for persistent reduction of the LIS by 0.5, was 1.60 ± 0.267 days in the HES group and 2.30 ± 0.578 days in the NS group (*P* = 0.348) (Figure 3). The intraoperative oxygenation index was calculated as the product of mean airway pressure and FiO_2_, divided by PaO_2_ and multiplied by 100.Values increased from 3.41 ± 1.42 to 3.77 ± 1.34 in the HES group and decreased from 3.19 ± 0.99 to 3.10± 0.93 in the NS group (Figure 3). The serial P/F ratios, LIS and oxygenation index values, did not differ significantly between the two groups, but a trend was seen showing faster improvement in the HES group (Figures 2 - 4). Four patients in each group required postoperative mechanical ventilatory support, as they did not meet the extubation criteria. There was no difference found between the two groups with regard to; duration of ventilation, ICU stay and total hospitalization period. (*P* value = 0.234, 1.00, 0.301) (Table 1).

**Table 1.** Demographic Profile and Perioperative Data

	HES (130/0.42)	NS	*P* value
**Age, y, Mean ± SD[Table-fn fn3469]**	29.00 ± 6.56	29.10 ± 8.10	1.00
**Gender (Male: Female)**	8:2	9/1	0.531
**Unilateral : bilateral, No.**	8:2	9/1	0.531
**Poly : mono trauma**	2:8	2/8	1.00
**New injury severity score, Mean ± SD**	10.90 ± 3.75	11.0 ± 2.66	0.180
**Time from injury to admission, h, Mean ± SD**	20.80 ± 23.20	22.80 ± 22.81	0.909
**Time from injury to surgery, d, Mean ± SD**	3.10 ± 1.52	3.30 ± 1.49	0.727
**Type of surgery (ILN:ORIF), n**	6:4	7:3	-
**Time of reaming, min, Mean ± SD**	38.50 ± 5.30	37.50 ± 4.25	0.691
**Duration of anesthesia, h, Mean ± SD**	129.00 ± 22.83	144.00 ± 27.16	0.217
**Surgery time, h, Mean ± SD**	116.00 ± 19.55	128.50 ± 25.83	0.300
**Mean blood pressure, mmHg, Mean ± SD**	85.20 ± 1.63	81.01 ± 1.63	0.088
**Heart rate, bpm, Mean ± SD**	91.08 ± 4.27	94.27 ± 4.27	0.604
**Duration of ventilation, d, Mean ± SD**	3.25 ± 1.25 (n=4)	3.67 ± 0.57 (n=3)	0.459
**Length of ICU stay, d, Mean ± SD**	5.00 ± 1.15 (n=4)	4.00 ± 0.00 (n=2)	0.268
**Hospitalization period, d, Mean ± SD**	7.50 ± .43 (n=10)	8.77 ± 3.07 (n=9)	0.481

Abbreviations: HES, hydroxyethyl starches; NS, normal saline, ICU, intensive care unit; ILN, interlock nailing; ORIF, open reduction and internal fixation

There were no anaphylactic reactions, HES-induced pruritus or central venous cannulation related complications (pneumothorax, carotid puncture or thromboembolic phenomenon). Wound infections requiring debridement/reoperation did not occur. None of the patients was readmitted to the hospital within 30 days after discharge. There were no mortalities.

## 4. Discussion

Surgery is known to elicit a stress response of combined endocrine and inflammatory origin. It can affect major organ functions including alveolar gas exchange and overall clinical outcomes (secondary insult) (10). Various pharmacological and surgical techniques have been developed to block this inflammatory cascade of events and to reduce the intravasation of fat during surgery (1). Well balanced volume therapy is known to; augment intravascular volume, maintain stable hemodynamics and improves microcirculatory organ perfusion (11). Hillebrecht noticed that the functional residual capacity decreased by 10% and diffusing capacity by 6% in healthy volunteers with a 22 m/kg saline infusion (12). In another randomized study, pulmonary morbidity was exceptionally high with 6 liters of crystalloids over 24 hours, as 10% of the patients developed respiratory failure (13). Thus, crystalloids alone have limited volume stabilizing effects, because only 20% of these substancesare found in intravascular spaces (14). Larger quantities, administered to compensate for interstitial loss, can contribute to the development of pulmonary edema, atelectasis, pneumonia, respiratory failure or hyperchloremic acidosis (12-14). Synthetic colloids produce more sustained intravascular volume expansion due to a high reflection coefficient and improved microcirculation (4, 15). In an animal model of sepsis, greater capillary luminal area, with less endothelial swelling and less parenchymal injury, was found with a colloid infusion compared to ringer lactate (2, 3). These products also result in lower perioperative blood loss, and red blood cell (RBC) transfusion due to relatively little interference with hemostasis (16,17). In the present study, young patients (mean age = 29 years) were enrolled,but associated thoracoabdominal or neurological injuries were excluded, because these could have affected the course of treatment. To optimize fluid replacement, intraoperative fluids were titrated according to central venous pressure measurement (CVP 12 + 2 mmHg) and hourly urine output. Total volume of fluid infused in the NS group in the present study was significantly more compared to the HES group (P < 0.05). However, organ dysfunction in terms of renal or respiratory compromise was not noticed in the saline group, this is probably because all the participants were otherwise healthy adults with normal reserves. In this trial, better oxygenation was anticipated in the HES group as a result of improved microcirculation. A trend, showing relatively faster improvement in the P/F ratio and early reduction in LIS scores in the HES group was noticed, but these values were not observed to be statistically relevant. Similarly, intraoperative oxygenation index values were found to be relatively higher in the HES group, but these values were well within the normal range in both groups. Avoidance of acid-base alterations by choosing an appropriate fluid regimen beneficially influences organ perfusion. In a study, on ICU patients, the base excess predicted outcomes by identifying patients who had a high risk of mortality. None of the patients in this study had acidotic changes. Undue postoperative nausea and vomiting did not occur in either group. No patient required reoperation for wound infections. There was no anaphylactic reaction, or CVP catheter related iatrogenic complications like pneumothorax, carotid puncture or thromboembolic episodes. However, the probability of serious adverse drug reactions (ADR) is less than 1% with HES (18), nevertheless, a larger number of patients are required to address these safety issues (19). Recognizing these limitations, only 20 patients were admitted during the study period with significant posttraumatic hypoxemia and SS > 5 were evaluated. Stable physiological parameters and a CVP of > 10 mmHg was ensured prior to the induction of anesthesia. Previous retrospective and prospective trials have shown superior outcomes in terms of the length of ventilator dependence, ICU stay, and duration of hospitalization, when surgical stabilization was done within 24 hours of the injury (1, 17). It has been suggested that definitive surgery should be avoided from day two to four post-injury, to allow for the stabilization of various inflammatory markers (20). Another school of thought is that in stable, appropriately resuscitated patients, early, but not emergent, definitive long bone fixation should be performed (1). Thus, the debate is still continuing (21). All the participants in this trial had hypoxemia and were operated 30-100 hours post-injury. Respiratory parameters improved in all of these patients after surgery. The duration of ICU stay, and the hospitalization period did not differ significantly between the two groups, and there was no mortality. To conclude, in this preliminary investigation of acute femur injury patients, with significant posttraumatic hypoxemia, both 6% HES (130/0.4) and 0.9% normal saline were found to be safe and effective intraoperative plasma volume substitutes. A trend showing a relatively faster improvement in P/F ratios and an early reduction in LIS values was observed in the HES group. Conducting a similar study with larger numbers of acute poly trauma patients presenting with posttraumatic hypoxemia, is expected to highlight the benefits and risks associated with colloid supplementation in this group of patients.
